# D-Xylose Blocks the Broad Negative Regulation of XylR on Lipid Metabolism and Affects Multiple Physiological Characteristics in Mycobacteria

**DOI:** 10.3390/ijms24087086

**Published:** 2023-04-11

**Authors:** Kun Wang, Xujie Cui, Xiaocui Ling, Jiarui Chen, Jiachen Zheng, Yuling Xiang, Weihui Li

**Affiliations:** State Key Laboratory for Conservation and Utilization of Subtropical Agro-Bioresources, College of Life Science and Technology, Guangxi University, Nanning 530004, China

**Keywords:** D-xylose, transcription regulation, mycobacteria, lipid metabolism, biofilm formation

## Abstract

D-xylose is the most abundant fermentable pentose, which usually represents an architectural component of the bacterial cell wall. However, its regulatory function and the involved signaling pathway in bacteria remain largely unclear. Here, we show that D-xylose can act as a signaling molecule to regulate the lipid metabolism and affect multiple physiological characteristics in mycobacteria. D-xylose directly interacts with XylR and inhibits its DNA-binding ability, thus blocking XylR-mediated repression. The xylose inhibitor, XylR, plays a global regulatory role and affects the expression of 166 mycobacterial genes that are involved in lipid synthesis and metabolism. Furthermore, we show that the xylose-dependent gene regulation of XylR affects the multiple physiological characteristics of *Mycobacterium smegmatis*, including bacterial size, colony phenotype, biofilm formation, cell aggregation, and antibiotic resistance. Finally, we found that XylR inhibited the survival of *Mycobacterium bovis* BCG in the host. Our findings provide novel insights into the molecular mechanism of lipid metabolism regulation and its correlation with bacterial physiological phenotypes.

## 1. Introduction

D-xylose is the most abundant fermentable pentose, and bacteria usually utilize it as a carbon and an energy source [1,2,3,4,5]. The xylose regulator, XylR, is a conserved transcription factor that is involved in the regulation of xylose’s metabolism in many bacterial species [5,6,7]. Generally, XylR functions as a negative regulator in the absence of the inducer xylose [5,8,9,10,11]. Recently, XylR was found to play a positive regulatory role in *Escherichia coli*, and the crystal structure of the XylR and D-xylose complex has been solved [6,7]. Strikingly, xylose has been detected in the hydrolysis products of the extracellular and surface-exposed materials in *M. smegmatis* [12], demonstrating that xylose is an architectural molecule of the cell wall in the bacterium. MSMEG_6021 (XylA) is a xylose isomerase involved in xylose metabolism; MSMEG_6018 (XylH), MSMEG_6019 (XylF), and MSMEG_6020 (XylE) are associated with the transport of xylose in *M. smegmatis*. Recently, it was found that D-xylose could act as a signaling molecule and could directly bind to XylR in several bacterial species [5,6,7]. Compared with the repressive effect on XylR in *Caulobacter crescentus* [5], D-xylose stimulates the positive regulation of XylR in *E. coli* [6,7]. Nevertheless, there are no reports on the effective molecules that regulate bacterial physiological or pathogenic function.

Mycobacteria are composed of a class of important bacterial species, including the pathogen of tuberculosis (TB) *M. tuberculosis* [13], and the fast-growing nonpathogenic model species, *M. smegmatis* [14]. As a model strain, *M. smegmatis* can be used to research various physiological processes in mycobacteria, including fatty acid metabolism, drug resistance, and gene regulatory networks [15]. Mycobacteria possess extraordinarily thick cell walls containing unique long-chain lipids, which are covalently linked with arabinogalactan through ester bonds [16,17,18]. Interestingly, these unique cell wall lipid complexes clearly contribute to mycobacterial biofilm formation [19,20,21,22], and the regulation of lipid metabolism genes is related to the mycobacterial antibiotic-resistant phenotype [23]. However, the molecular mechanism underlying the regulation of extracellular lipid metabolism by the D-xylose molecule and its impact on physiological characteristics in mycobacteria are largely unclear. Here, we reported that D-xylose could act as a signaling molecule and coordinate with XylR to regulate bacterial lipid metabolism in *M. smegmatis*. XylR functions as a negative regulator and broadly inhibits the expression of a large number of genes, including 166 genes involved in lipid synthesis and metabolism in *M. smegmatis*. D-xylose could directly bind to the *M. smegamtis* XylR and block its negative regulation of the lipid metabolism, which finally affected multiple physiological characteristics of mycobacteria, including bacterial size, colony phenotype, biofilm formation, cell aggregation, and drug resistance. As far as we know, this is the first report on the regulatory effects of the D-xylose molecule on key bacterial physiological functions through the xylose operon.

## 2. Results

### 2.1. XylR Negatively Regulates the Expression of Its Own Gene Operon

**Figure 1:**
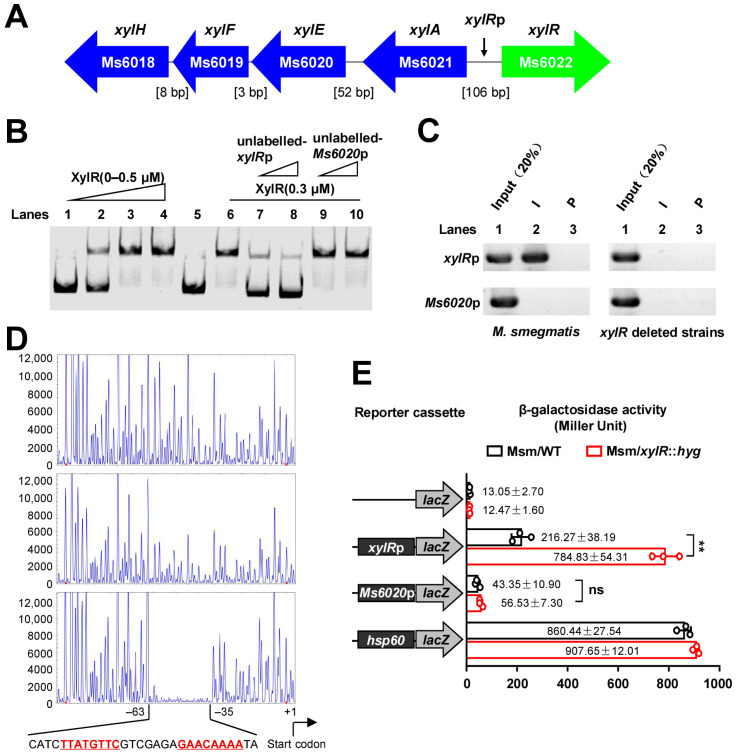
Detection of XylR regulatory activity and identification of its DNA-binding motif. (**A**) The *xylR* and D-xylose metabolism gene cluster *Ms6021*–*18* is located on the opposite of the genome. Additionally, they shared a common promoter, *xylR*p, indicated by the black arrow. (**B**) EMSA assays for the specific DNA-binding activity of XylR. FITC-labeled promoter DNA substrate was co-incubated with different concentrations of XylR protein (lanes 1–4). The DNA-binding activity of XylR was examined with negative control of *Ms6020*p (lane 5). The ability of unlabeled *xylR*p (lanes 7–8) and unlabeled *Ms6020*p (lanes 9–10) to compete with binding of the labeled *xylR*p with XylR protein was examined. (**C**) ChIP assays. ChIP using pre-immune (P) or immune sera (I) raised against XylR. The DNA *Ms6020*p was used as a negative control. (**D**) DNaseI footprinting experiments. Protection of the *xylR*p against DNaseI digestion by increasing amounts of XylR (0 μM, 1.5 μM and 3.0 μM) was evaluated. The sequences of the protected regions on the coding strand are underlined. (**E**) The assays of β-galactosidase activity. The effect of XylR on the gene expression was assayed by constructing a series of *lacZ* alone or promoter-*lacZ* co-expression plasmids. These plasmids were transformed into Msm/WT and Msm/*xylR*::*hyg* strains. The activity of β-galactosidase was further examined and presented as Miller units (right panel). Left column: schematic representation of each clone used to generate recombinant strains. Null promoter-*lacZ*, *Ms6020*p-*lacZ*, and *hsp60*-*lacZ* were used as controls. Right column: β-galactosidase activity was expressed as Miller units. The values presented were the averages of three independent experiments. For statistical analysis, two-way analysis of variance with Bonferroni multiple comparison tests was performed using a *P*-value of <0.01. The *P*-values of the results (<0.01) are indicated by two asterisk (**) on top of the column in the figure.

To explore the regulatory function of XylR, we examined the potential binding of XylR to the upstream region of the D-xylose metabolism gene cluster within the *xylR* local gene cluster in *M. smegmatis* (Figure 1A). The intergenic sequence, *xylR*p (as indicated by arrows in Figure 1A), within the *xylR* local gene cluster was used to test the interaction between it and XylR through previously reported bacterial one-hybrid assays [24]. Only the *xylR*+*xylR*p and *xylR*+*Ms6021*p co-transformant strains grew well on the screening medium (Figure S1A). We further used an electrophoretic mobility shift assay (EMSA) to confirm the binding of the XylR protein to the upstream region of the operon in vitro. As shown in Figure 1B, when 50 ng of upstream DNA substrates (*xylR*p) were co-incubated with increasing amounts of XylR (0 μM, 0.1 μM, 0.3 μM, and 0.5 μM), clear shifted bands were observed (Figure 1B, lane 1 to lane 4). A competition assay confirmed the specificity of XylR binding to its promoter DNA. An unlabeled specific *xylR*p or unspecific *Ms6020*p DNA substrate was used to compete with the labeled *xylR*p. As shown in Figure 1B, an unlabeled *xylR*p DNA substrate (Figure 1B, lane 7 and lane 8), but not an *Ms6020*p DNA substrate (Figure 1B, lane 9 and lane 10), could competitively inhibit the binding of XylR to the labeled upstream DNA of *xylR* operon.

Further chromatin immunoprecipitation (ChIP) assays also confirmed the binding of XylR to the upstream DNA of *xylR* operon in vivo. As shown in Figure 1C, in the left panel, XylR could be cross-linked with the upstream DNA *xylR*p in *M. smegmatis*. The promoter DNA could be recovered by immunoprecipitation with XylR antiserum (Figure 1C, left lane 2). By contrast, the pre-immune serum failed to precipitate significant amounts of DNA (Figure 1C, left lane 3). In addition, *Ms6020*p could not be recovered with XylR antiserum. The experiment was also performed in an *xylR*-deleted strain that was used as the negative control (Figure 1C, right panel). These findings strongly suggested that XylR could bind to its local gene cluster promoter region both in vitro and in vivo in mycobacteria.

Subsequently, we characterized the DNA-binding motif of XylR by DNase I footprinting assays. As shown in Figure 1D, when increasing amounts of XylR protein (0–3 μM) were co-incubated with DNase I, the region around CATCTTATGTTCGTCGAGAGAACAAAATA was obviously protected on the coding strand. The protected DNA region extended from position −63 to −35 in the DNA strand (Figure 1D). A palindromic motif formed by two inverted repeats that were partially matched, and were separated from each other by seven nucleotides, was found in this region. Further, EMSA assays confirmed the significance of the motif for specific recognition by XylR. DNA substrate mutants were synthesized (Figure S2A), and EMSA assays were conducted (Figure S2B,C). Therefore, XylR recognizes a palindromic motif, and TTATGTTC---GAACAAAA is the most valuable nucleotides motif.

Next, we determined the specific effect of the regulatory function of XylR in vivo using β-galactosidase as a reporter gene. As shown in Figure 1E, a series of promoter-*lacZ* reporter plasmids were constructed. Notably, the expression of *lacZ* was significantly up-regulated in the *xylR*-deleted mutant (Figure 1E), and remarkably down-regulated in the *xylR*-overexpression strain (Figure S3) compared with the wild-type *M. smegmatis* strain. However, there was no significant difference between the wild-type and mutant in the expression of *lacZ* when an irrelevant control, *Ms6020*p, was used as a promoter. These results suggested that XylR could inhibit the expression of its own gene.

Taken together, our data indicate that XylR acts as an inhibitor and negatively regulates *xylR* operon expression.

### 2.2. D-Xylose Inhibits the DNA-Binding Activity of XylR and Effectively Counteracts the Negative Regulation of XylR

**Figure 2:**
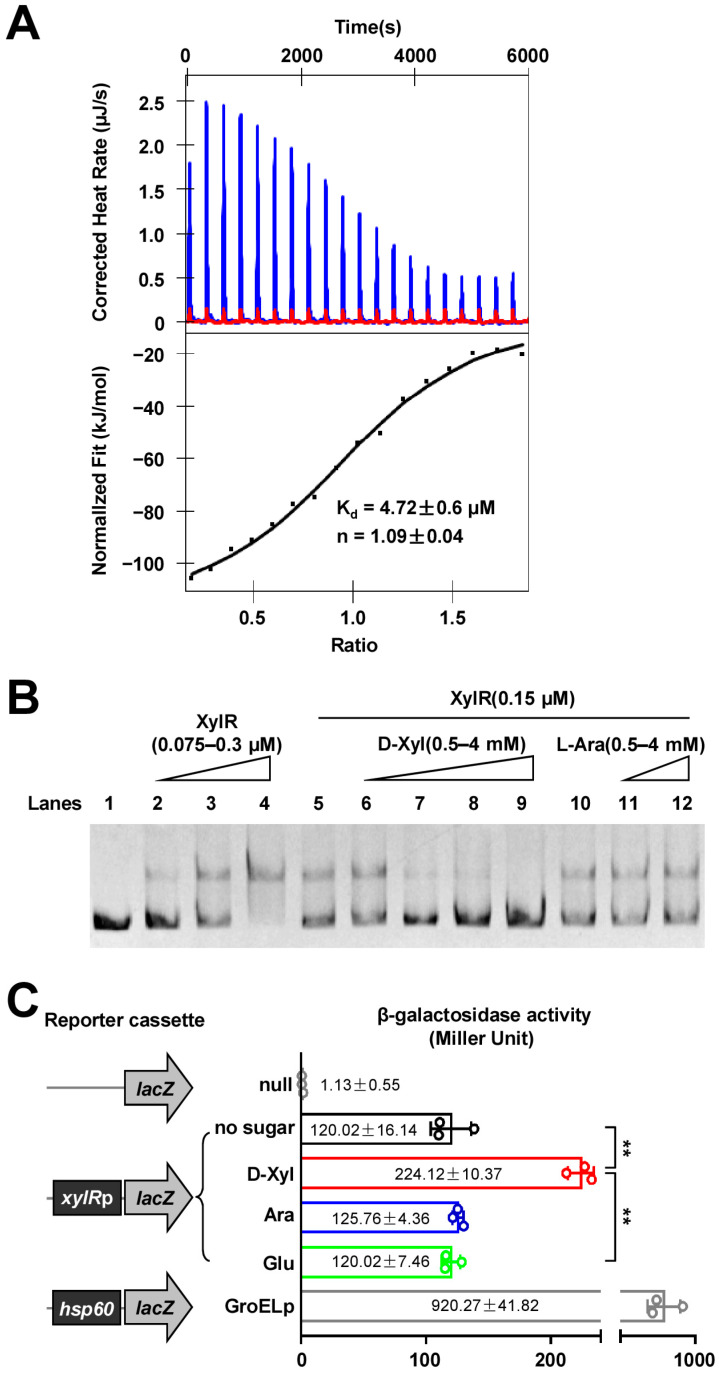
Assay for the interaction between D-xylose and XylR. (**A**) ITC assays for the physical interaction of D-xylose with the XylR protein. Original titration data and integrated heat measurements are shown in the upper and lower plots, respectively. The solid line in the bottom panel represents the best fit to a one-site binding model of the interaction of XylR with D-xylose. (**B**) EMSA assays for the effect of D-xylose on DNA-binding activity of XylR. *xylR*p was co-incubated with increasing amounts of XylR protein (lanes 1–4). The concentration of XylR was immobilize and then increasing amounts of D-xylose (0.5–4 mM) (lanes 6–9) or L-Ara (0.5–4 mM) (lanes 11–12) were added into the reactions. (**C**) β-galactosidase activity assays. The effect of D-xylose on the genes expression was assayed by constructing *xylR*p-*lacZ* plasmid. The activity of β-galactosidase was further examined in the wild-type *M. smegmatis* strain (Msm/WT) and the data were presented as Miller units (right panel). Left column: schematic representation of each clone used to generate recombinant strains. Null promoter-*lacZ* and *hsp60*-*lacZ* were used as controls. The D-xylose was added into 7H9 medium when the strains were cultured. The no sugar, L-Ara and D-Glu were used as controls. The values presented were the averages of three independent biological experiments. For statistical analysis, two-way analysis of variance with Bonferroni multiple comparison tests was performed using a *P*-value of <0.01. The *P*-values of the results (<0.01) are indicated by two asterisks (**) on top of the column in the figure.

Next, we used isothermal titration calorimetry (ITC) to examine the potential interaction between the XylR and D-xylose molecules. Figure 2A showed the raw data for titration of D-xylose against XylR, indicating that the binding stoichiometry between D-xylose and XylR was 1:1 (*n* = 1.09 ± 0.04), and the binding affinity of the interaction (Kd) was 4.72 ± 0.6 μM. By contrast, we did not detect an interaction between XylR and L-arabinose, which are used as the negative control molecules for the assay, under similar experimental conditions (Figure S4). Thus, D-xylose could specifically interact with XylR.

The physical interaction between D-xylose and XylR implies that D-xylose would have a regulatory effect on the activity of XylR. We then used EMSA to confirm this hypothesis. As shown in Figure 2B, when increasing the amounts of XylR in the reactions (lanes 1–4), a stepwise increase in the amount of the shifted DNA was observed, indicating that XylR could bind to the upstream DNA fragment of *xylR* well. The addition of increasing amounts of D-xylose (0.5–4 mM) led to a corresponding decrease in the amounts of shifted DNA substrates (Figure 2B, lanes 6–9), indicating that D-xylose inhibits the activity of XylR in a concentration-dependent manner. In contrast, we did not detect a significant effect of the negative control molecule, L-arabinose, on the DNA-binding activity of XylR (Figure 2B, lanes 11–12). These results indicated that D-xylose specifically inhibited the ability of XylR to bind to its target DNA.

Next, we determined the specific effect of D-xylose on the regulatory function of XylR in vivo using β-galactosidase as the reporter gene. As shown in Figure 2C, when adding 10 mM each of D-xylose, L-arabinose, and D-glucose, respectively, into the medium, only D-xylose was shown to be capable of significantly inducing the expression of *lacZ* in the wild-type *M. smegmatis* strain (Figure 2C). These results strongly suggested that D-xylose could specifically neutralize the negative regulation of XylR in *M. smegmatis*.

Therefore, our findings indicate that D-xylose could effectively counteract negative regulation of XylR.

### 2.3. XylR Is a Negative Regulator and Broadly Affects the Expression of Lipid Metabolism Genes in M. smegmatis

**Figure 3:**
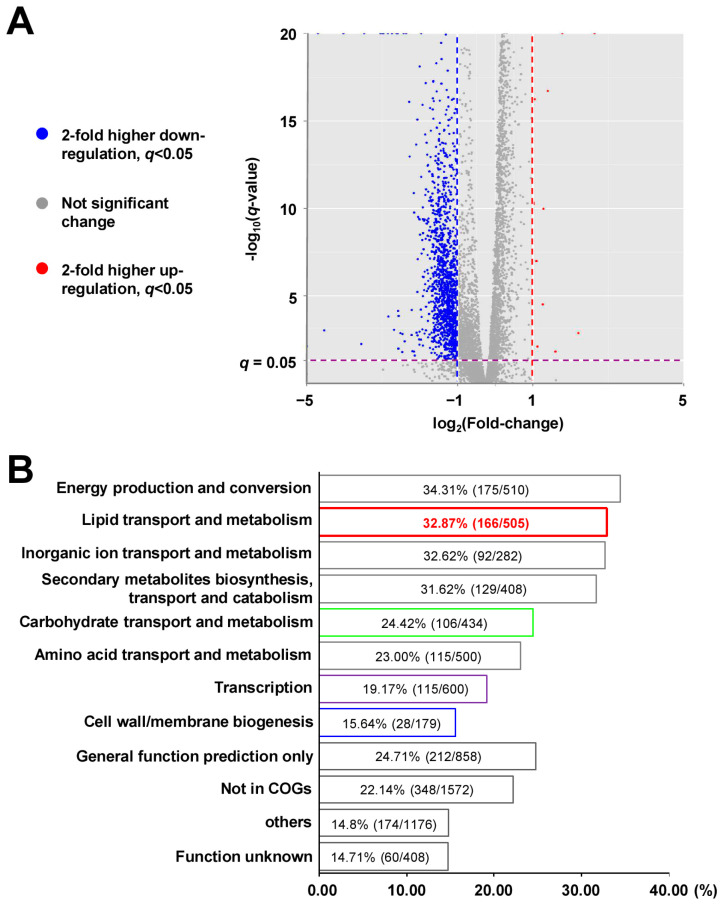
Transcriptomic assays for the effect of *xylR* on the gene expression of *M. smegmatis*. (**A**) The volcanic diagram shows gene expression differences between Msm/pMV261 and Msm/pMV261-*xylR* strains through transcriptomic analysis. The blue spots show that genes are significantly down-regulated. The red and gray spots show significantly up-regulated genes and not significantly changed genes, respectively. (**B**) Functional categories of 1494 significantly down-regulated target genes of XylR in *M. smegmatis*. Classification of the target genes was analyzed in terms of COG categories.

To explore the potential regulatory function of XylR in *M. smegmatis*, we investigated its effect on gene expression using a transcriptomic analysis. As shown in Figure 3A, approximately 1500 genes in the *xylR*-overexpressed strain were found to be significantly down-regulated when compared with those of the wild-type strain transformed with empty plasmid pMV261 (wild-type strain) (Supplementary Table S1). Notably, a large number of lipid transport and metabolism genes (166) were identified, which accounts for about 32.87% of this type of gene in the *M. smegmatis* genome (Figure 3B). This percentage is only slightly less than the energy production and conversion gene (34.31%) among all these down-regulated genes.

These results suggest that XylR acts as a negative regulator and broadly affects the expression of mycobacterial lipid metabolism genes.

### 2.4. XylR Negatively Regulates the Synthesis of Lipid in M. smegmatis

**Figure 4:**
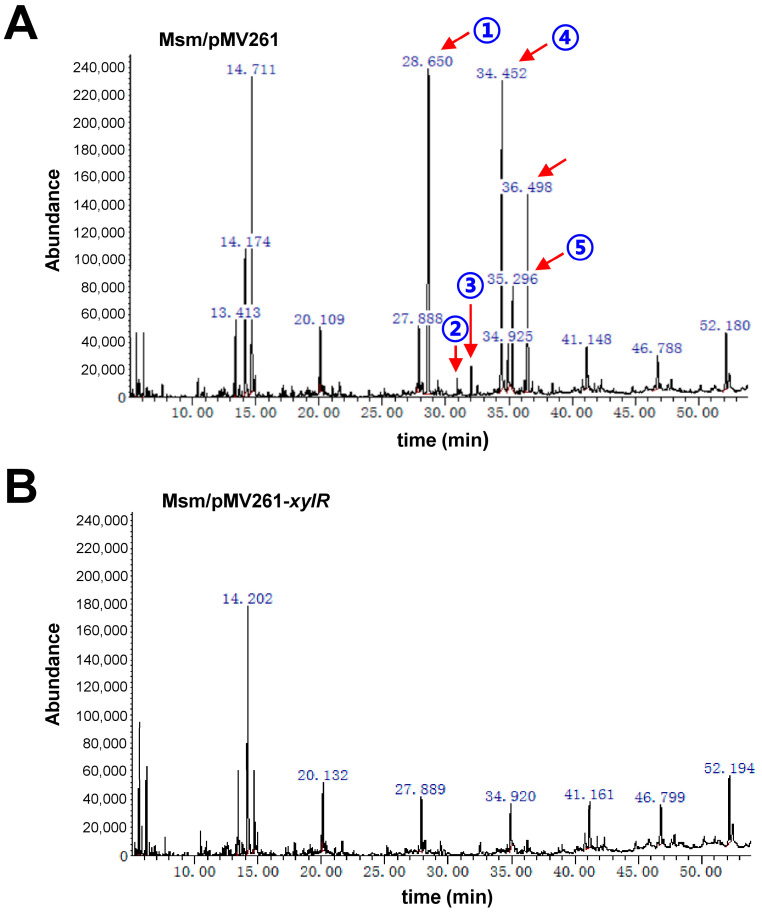
GC/MS assays for detecting the lipids of *M. smegmatis* strain. The six lipids identified with significant changes are indicated by arrows. The levels of lipids in the *xylR-*overexpression strain (**B**) were lower than in the Msm/pMV261 strain (**A**).

Lipids represent essential components of the mycobacterial cell wall. XylR broadly affects the expression of lipid transport and metabolism genes, suggesting that XylR could be involved in the regulation of cell wall lipid synthesis in *M. smegmatis*. In order to test this possibility, we used GC/MS to detect the contents of cell wall lipids in the *xylR*-overexpression strain and the wild-type strain. Interestingly, the contents of cell wall lipids in the *xylR-*overexpression strain were found to be obviously less than that of the wild-type strain (Figure 4A,B), and the lipids with significant differences were marked in Figure 4A ((1): Hexadecanoic acid methyl ester, (2): Acetic acid ethyl ester, (3): Tridecanoic acid methyl ester, (4): 9-Octadecenoic acid methyl ester, (5): Octadecanoic acid methyl ester, and (6): Nonadecanoic acid methyl ester, respectively).

These results confirmed the inhibitory effect of XylR on cell wall lipid synthesis in *M. smegmatis.*

### 2.5. XylR Regulates Bacterial Size, Colony Phenotype, and Biofilm Formation in M. smegmatis

**Figure 5:**
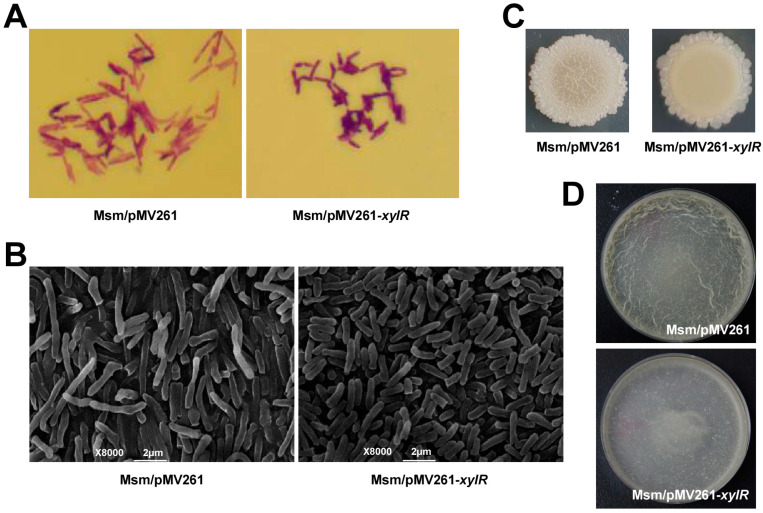
Effects of XylR on bacterial size, colony morphology, and biofilm formation of *M. smegmatis*. (**A**) The observation of *M. smegmatis* with a light microscope after staining with 2% crystal violet. (**B**) Scanning electron microscopy assays of cell morphology. The images were taken at 8000× magnification. (**C**) Representative colony morphology of Msm/pMV261 (left panel) and Msm/pMV261-*xylR* (right panel). All recombinant mycobacterial strains were spot and grown on 7H10 medium. (**D**) The difference in surface morphology of the biofilm at air-liquid interface between the Msm/pMV261 (the upper panel) and Msm/pMV261-*xylR* (the lower panel) strains. All recombinant mycobacterial strains were grown in M63 medium.

XylR inhibits the synthesis of cell wall lipids in *M. smegmatis*, implying that this regulator could affect multiple bacterial physiological characteristics. To test this hypothesis, we first stained the recombinant strain with crystal violet, and the bacterial length of the *xylR-*overexpression strain became significantly smaller and its staining was aggravated (Figure 5A). A scanning electron microscope also showed that the length of the *xylR*-overexpression strain was smaller than that of the wild-type strain (Figure 5B). Next, an observation of the colony morphology showed that the wild-type strain presented a typical wrinkled surface. In contrast, the *xylR*-overexpression strain showed a relatively smooth surface and lacked a wrinkled surface (Figure 5C). These results suggested that XylR could regulate the colony phenotypes of *M. smegmatis*. Further, as shown in Figure 5D, compared with the significantly wrinkled pellicles biofilm of the wild-type strain, the biofilm of the *xylR*-overexpression strain was thinner, indicating a negative regulation of XylR on bacterial biofilm formation.

Therefore, our results suggest that XylR could regulate the bacterial size and colony phenotype and negatively regulate biofilm formation in *M. smegmatis*.

### 2.6. D-Xylose Has a Contrary Regulatory Role to XylR for the Colony Phenotype and Biofilm Formation in M. smegmatis

**Figure 6:**
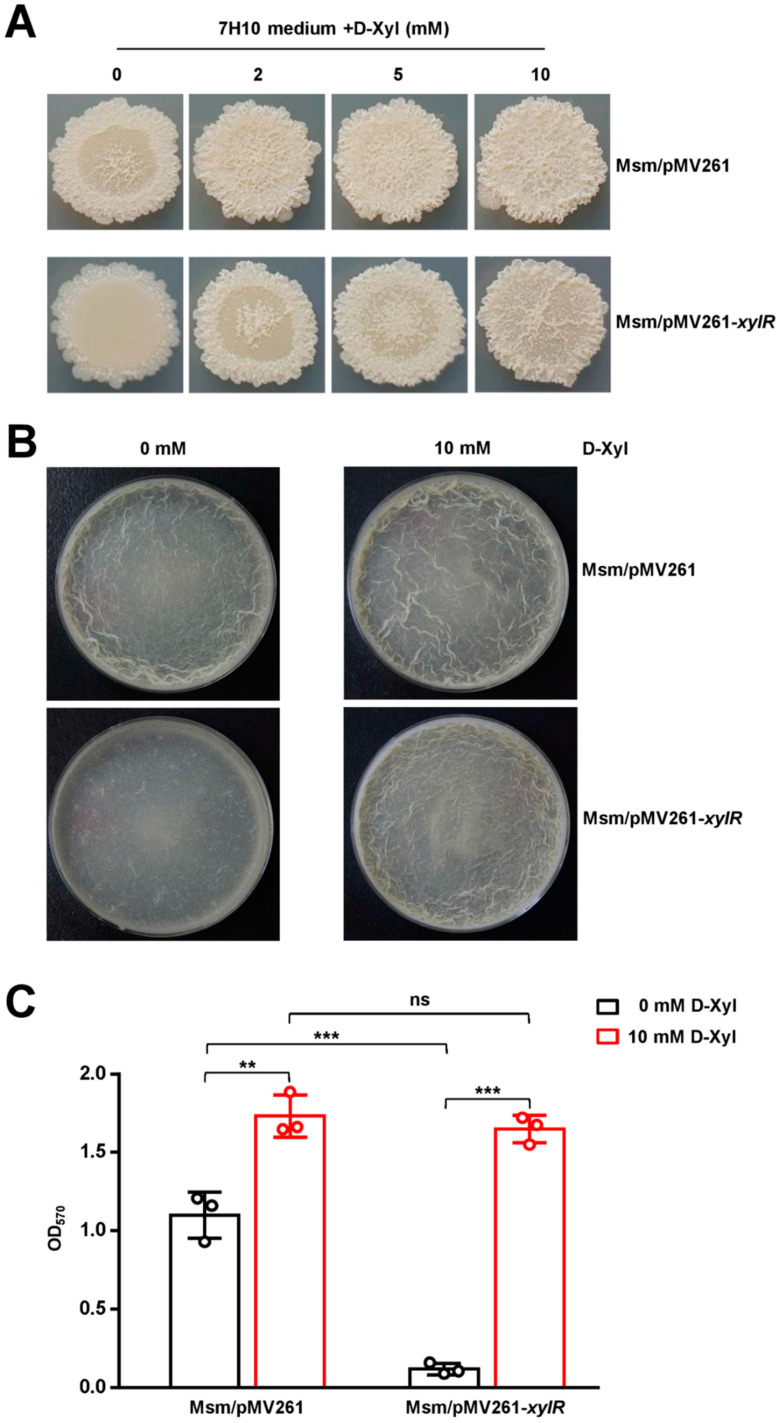
Assays for the regulatory effects of D-xylose on the wild-type and *xylR*-overexpression strains. (**A**) Representative colony morphology of the recombinant *M. smegmatis* strains spot on 7H10 medium. The Msm/pMV261 (the upper panel) and Msm/pMV261-*xylR* (the lower panel) strains were grown on solid medium 7H10 containing the increasing amounts of D-xylose (0, 2, and 5, 10 mM). (**B**) The effect of D-xylose on the biofilm formation of two recombinant *M. smegmatis* strains. The Msm/pMV261 (the upper panel) and Msm/pMV261-*xylR* (the lower panel) strains were grown in M63 medium containing the increasing amounts of D-xylose (0 and 10 mM). (**C**) Quantitation of biofilm formation by crystal violet (CV) staining. Tests were performed in three biological replicates, and error bars indicate the standard variations. Statistical analysis was carried out using multiple Student’s *t*-tests. The *P*-values of the results (<0.01, <0.001) are indicated by asterisk (**, ***) on top of the column in the figure. Not significant change (*P* > 0.05) is indicated by ns.

Next, we first examined the regulatory effects of D-Xylose on the colony phenotype of *M. smegmatis*. As shown in Figure 6A (upper panel), the wild-type strain showed a typical rough-edged colony and wrinkled surface. Interestingly, when providing an increasing concentration of D-xylose (2–10 mM) in the 7H10 medium, a corresponding increase in the rough-edged colony and wrinkled surface was clearly observed, indicating that D-xylose stimulated the formation of the rough-edged colony phenotype. We further compared the effects of D-Xylose on the phenotype of the *xylR*-overexpression strain with that of the wild-type strain. Interestingly, the *xylR*-overexpression strain produced a rough-edged colony at a slower rate of increase (Figure 6A, lower panel), followed by increasing amounts of D-xylose, indicating that the *xylR* overexpression could effectively counteract the stimulatory role of D-xylose on the phenotype formation of *M. smegmatis*.

To further verify the regulatory effect of D-xylose on the biofilm formation in *M. smegmatis,* we performed the air-liquid surface biofilm observations and the biofilm quantification. As shown in Figure 6B, excessive D-xylose could stimulate the production of wrinkled pellicles at the air–liquid interface in wild-type and *xylR*-overexpressing strains in the M63 medium. Subsequent biofilm quantification showed similar results (Figure 6C), indicating the positive regulation of D-xylose on bacterial biofilm formation.

Therefore, our results suggest that XylR has a contrary regulatory role to D-xylose for the colony phenotype and biofilm formation of *M. smegmatis*.

### 2.7. D-Xylose Can Effectively Counteract xylR-Dependent Drug Resistance and Cell Aggregation

**Figure 7:**
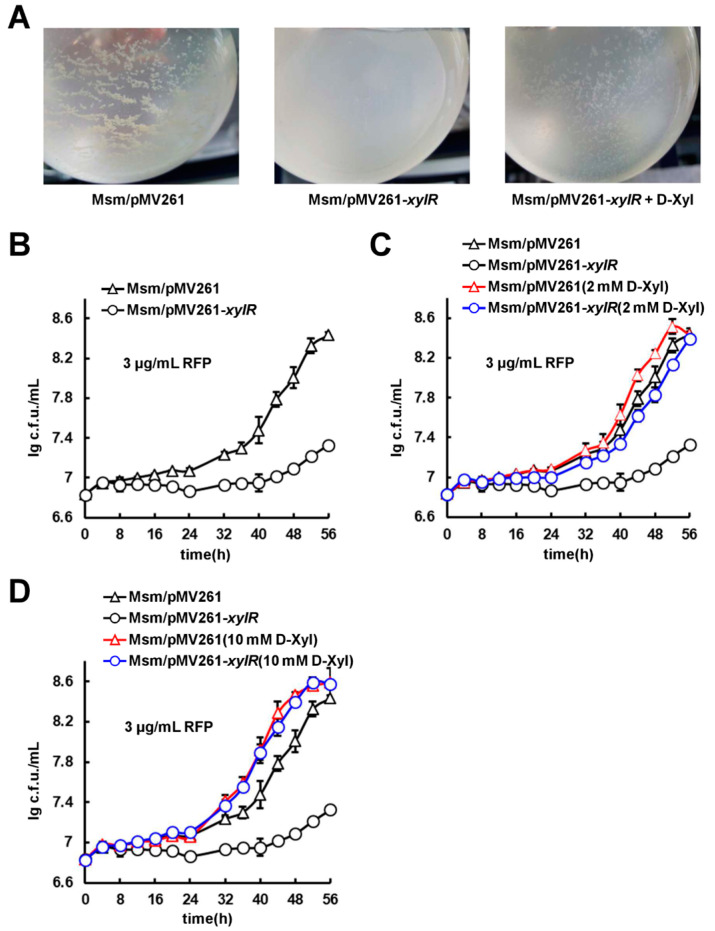
Assays for the regulation of D-xylose on *xylR*-dependent cell aggregation and drug resistance. Growth curves of the wild-type and *xylR*-overexpression strains were determined as described in Section 4. (**A**) Assays for the effect of D-xylose on *M. smegmatis* cell aggregation. Msm/pMV261 (left panel), Msm/pMV261-*xylR* (middle panel), and Msm/pMV261-*xylR* (10 mM D-xylose) (right panel) strains were grown in 7H9 medium in the presence of 30 μg/mL INH. The pictures of cell aggregates and particles formation were photographed by camera. (**B**) The effect of *xylR* overexpression on mycobacterial antibiotic resistance. Msm/pMV261 and Msm/pMV261-*xylR* strains were grown in 7H9 medium supplemented with 3 μg/mL RFP. The effect of D-xylose on the resistant phenotype of *xylR*-expression. Msm/pMV261 and Msm/pMV261-*xylR* strains were grown in 7H9 medium containing 3 μg/mL RFP and in the presence of 2 mM D-xylose (**C**) or 10 mM D-xylose (**D**). The D-xylose could efficiently neutralize the *xylR* overexpression-dependent resistant phenotype in *M. smegmatis.* Representative growth curves are shown. Error bars represent the variant range of the data derived from three biological replicates.

We further investigated the effects of *xylR* on mycobacterial cell aggregation. Interestingly, when adding 30 µg/mL INH drug into the medium as a stress inducer, the wild-type strain, but not the *xylR*-overexpression strains, were found to form cell aggregates (Figure 7A). However, when 10 mM D-xylose was further supplemented into the medium, cell aggregates were clearly observed in the *xylR*-overexpression strain, which were very similar to those of the wild-type strain (Figure 7A). This suggests that D-xylose could perfectly neutralize the role of *xylR* overexpression on cell aggregation. Therefore, overexpression of xylR inhibited cell aggregations, and the addition of D-xylose in the medium could obviously rescue the growth phenotypes.

Since the lipid synthesis of the mycobacterial cell wall is usually correlated with its drug-resistant phenotype, we investigated the effect of both *xylR* overexpression and D-xylose on mycobacterial antibiotic resistance. As shown in Figure 7B, compared with the wild-type strain, the *xylR*-overexpression strain grew more slowly in the 7H9 medium containing 3 μg/mL of rifampicin (RFP). Additionally, there was no obvious difference in the growth of the wild-type and *xylR*-overexpression strains in the absence of antibiotics (Figure S5A). These results suggest that *xylR* negatively regulates the RFP-resistance of *M. smegmatis*.

We then determined the effect of D-xylose on the resistant phenotype of *xylR* overexpression. As shown in Figure 7C, the growth difference between the *xylR*-overexpression strain and wild-type strain significantly reduced when 2 mM of D-xylose was further supplemented into the medium above. More obviously, the growth of the *xylR*-overexpression strain is almost identical to the wild-type strain with the addition of 10 mM of D-xylose (Figure 7D). Essentially, there was no obvious difference in the growth of the wild-type and *xylR*-overexpression strains in the presence of D-xylose (Figure S5B–E).

Therefore, D-xylose could effectively neutralize the *xylR*-dependent and resistant phenotype in *M. smegmatis.* These results are consistent with our other findings that D-xylose specifically targets the negative regulator *xylR* and counteracts its inhibition on the lipid metabolism.

### 2.8. XylR Attenuates M. bovis BCG Survival in the Host

**Figure 8:**
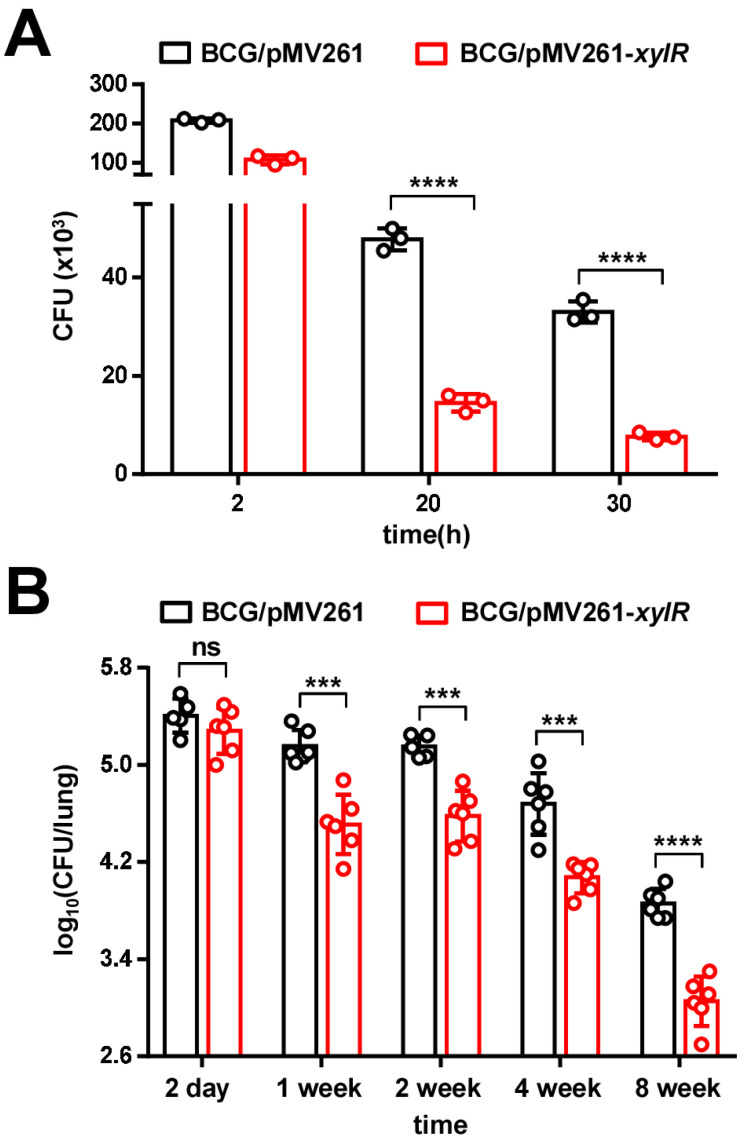
Assays for testing the effect of XylR on intracellular survival of mycobacteria in the host. (**A**) Detection of BMDMs intracellular survival efficiency infected with BCG/pMV261 strain and BCG/pMV261-*xylR* strain, respectively. Error bars represent the S.D. from three biological experiments. (**B**) Detection of the mycobacterial burdens in the lungs of C57BL/6 mice infected with the BCG/pMV261 strain and BCG/pMV261-*xylR* strain, respectively. Error bars represent the S.D. from six biological experiments. Statistical analysis was carried out using multiple Student’s *t*-tests. The *P*-values of the results (<0.001, <0.0001) are indicated by asterisk (***, ****) on top of the column in the figure. Not significant change (*P* > 0.05) is indicated by ns.

XylR inhibits lipid synthesis and negatively regulates biofilm formation and drug resistance in *M. smegmatis,* which implies that XylR could affect mycobacterial survival in the host. To confirm this, we used the *xylR*-overexpression strain (BCG/pMV261-*xylR*) and the wild-type strain (BCG/pMV261) to infect mouse bone marrow-derived macrophages (BMDMs). The results revealed that the intracellular survival efficiency of the BCG/pMV261-*xylR* strain in macrophages was significantly lower than that of the BCG/pMV261 strain 20 and 30 h post infection (hpi) (Figure 8A). These results suggested that XylR attenuates mycobacterial survival in macrophages.

Subsequently, we tested mycobacterial burdens in the lungs of C57BL/6 mice infected with the BCG/pMV261 strain and the BCG/pMV261-*xylR* strain, respectively. As shown in Figure 8B, the mycobacterial burdens in the lungs of the mice infected with these two strains showed no significant difference 2 days post infection (dpi). Nevertheless, the mycobacterial burdens in the lungs of the mice infected with the BCG/pMV261-*xylR* strain decreased significantly compared with that of the mice infected with the wild-type BCG strain from 1 to 8 weeks, which suggests that XylR inhibits the *M. bovis* BCG load in the lung of mice.

Taken together, our results confirm that *xylR* attenuates mycobacterial survival in vitro, including in macrophages and mice.

## 3. Discussion

D-xylose has been detected in the hydrolysis products of the extracellular and surface-exposed materials in the *M. smegmatis* [12]. In the present study, we have provided the first piece of evidence showing that D-xylose also acts as an important signaling molecule in *M. smegmatis*. D-xylose physically interacts with the global inhibitor XylR and blocks its negative regulation on lipid metabolism, which finally affects multiple physiological phenotypes of *M. smegmatis*. Therefore, we reported a novel D-xylose-triggered and XylR-dependent signaling pathway that regulates the lipid metabolism in *M. smegmatis*.

XylR has been previously reported as a regulator of xylose transport and metabolism and is conserved in multiple bacterial species. However, the physiological function of XylR remains largely unclear. In the present study, based on *xylR* overexpression and a knockout analysis, we confirmed that the transcriptional factor negatively regulated the expression of its own gene operon. More interestingly, the D-xylose molecule was shown to physically interact with the XylR protein and inhibit the DNA-binding activity of XylR. D-xylose could efficiently counteract all these *xylR*-dependent regulations on gene expression and physiological phenotypes. XylR was involved in the regulation of cell wall lipid metabolism in *M. smegmatis*. XylR was shown to broadly affect the expression of 166 lipid transport and metabolism genes. Notably, this regulation finally affected multiple physiological characteristics of *M. smegmatis*, including bacterial size, colony phenotype, biofilm formation, cell aggregation, and drug resistance. Additionally, XylR also inhibited the survival of *M. bovis* BCG in the host. Although it seems complicated for the exact *xylR*-dependent mechanism of metabolism regulation on lipids synthesis, the present study has clearly characterized XylR as a novel, negative regulator for the lipid metabolism, and D-xylose functions as an effector molecule for the regulation in *M. smegmatis*.

The lipid content of the mycobacterial cell wall accounts for more than half of its dry weight, and the genome sequence of *M. tuberculosis* shows that a large proportion of genes are devoted to lipid production or metabolism [13,25,26]. In the present study, we validated the negative regulation of XylR on the expression of lipid transport and metabolism genes, which might alter the lipid content of the cell wall in *M. smegmatis*. Consistently, the colony surface of the *xylR*-overexpression strain became quite smooth. As expected, the drug resistance of the recombinant strains was also found to change with the expression level of lipid metabolism genes in *M. smegmatis*. The *xylR*-overexpression strain was more sensitive to RFP compared with the wild-type strain. Most interestingly, D-xylose can neutralize the *xylR*-dependent negative regulation and trigger the formation of several typical phenotypes. For example, D-xylose can efficiently counteract xylR-dependent colony phenotype change, drug resistance, and cell aggregation. Therefore, our data support a model in which XylR can sense the xylose signal and broadly regulate the expression of lipid metabolism genes that further affect mycobacterial multiple physiological characteristics. To our knowledge, this is the first report on D-xylose acting as a signaling molecule to affect the important physiological functions of bacteria. Our findings provided new clues for further understanding the molecular mechanism of cell wall lipid metabolism regulation and its correlation with bacterial physiological phenotypes in mycobacteria.

## 4. Materials and Methods

### 4.1. Cloning, Expression, and Purification of Recombinant Proteins

*xylR* was amplified by polymerase chain reaction (PCR) using *M. smegmatis* genomic DNA as template, and the primer pairs are as follows: (Ms6022-F: GACGGAATTCCATTGGCAAACGGAACCCAG, Ms6022-R: ATATTCTAGATCAACCGGCCTCGACACCGC). All DNA primers were synthesized by Invitrogen (Wuhan, China). The amplified *xylR* gene fragments were cloned into the vectors pET28a or pMV261 to produce the recombinant plasmids. The *E. coli* BL21 (DE3) cells were collected, and proteins were purified by Ni^2+^ affinity columns as described in earlier reports [27].

### 4.2. Bacterial One-Hybrid Assays

Bacterial one-hybrid assays were carried out as described previously [24]. Co-transformants containing the pBX-Rv2031/pTRG-Rv3133 plasmids served as positive controls (CK^+^) and co-transformants containing the empty vectors pBX and pTRG served as negative controls (CK^−^).

### 4.3. Electrophoretic Mobility Shift Assay (EMSA)

DNA fragments for EMSA were amplified by PCR or synthesized by Invitrogen (Wuhan, China). The DNA-binding assays were performed according to a previously described procedure [23] with several changes. The reactions (20 μL) for measuring the mobility shift contained DNA fragments and various amounts of XylR protein diluted in a buffer containing 50 mM Tris-HCl pH 7.5, 10 mM MgCl_2_, 1 mM DTT, and 50 mM NaCl. The mixtures were co-incubated at room temperature for 30 min and then directly subjected to 5% native Polyacrylamide Gel Electrophoresis containing 0.5× Tris-borate-EDTA buffer. Electrophoresis was performed at 150 V. Images were acquired by Typhoon Scanner (GE Healthcare, Chicago, IL, USA).

### 4.4. Chromatin Immunoprecipitation Assay

Chromatin immunoprecipitation (ChIP) assay was used to detect the DNA-binding ability of XylR in vivo. *M. smegmatis* wild-type and *xylR*-deleted strains were cultivated in 100 mL 7H9 medium to log phase (OD_600_ ≈ 1.0). A total of 1% formaldehyde was used to fix the cells for 30 min. A total of 125 mM glycine was added and let stand for 5 min to stop the reaction, and then the cells were harvested. Cells were treated as described previously [28]. Samples of those were incubated with antibodies of XylR or pre-immune serum for 3 h at 4 °C. The complexes were treated as described previously [28]. The protocol of PCR amplification contained one denaturation step of 5 min at 95 °C, then 28 cycles of 1 min at 95 °C, 1 min at 60 °C, and 1 min at 72 °C.

### 4.5. Footprinting Assay

The *xylR*p promoter DNA was amplified with specific primers (the reverse primer labeled with Fluorescein Isothiocyanate (FITC)). The purified DNA fragment was added to the reaction mixture (containing various amounts of XylR) at room temperature for 30 min as in EMSA. All of the mixtures were treated with DNaseI (1 unit, Fermentas) (Fermentas China Co., Ltd., Shenzhen, China) at 37 °C for 2 min 30 s as described previously [23]. The results were analyzed with Applied Biosystems 37030XL DNA analyzer (manufactured by Tsingke Company, Wuhan, China).

### 4.6. β-Galactosidase Activity Assays

β-galactosidase activity experiments were performed in *M. smegmatis* based on the expression vector of pMV261 [29]. Target and control promoters were cloned into pMV261. As the reporter gene, *lacZ* was cloned behind the promoters. The plasmids were transformed into *xylR* mutant and wild-type *M. smegmatis* strain to obtain the corresponding recombinant reporter strains, respectively. All recombinant strains were grown in 7H9 medium at 37 °C until log phase. β-galactosidase measurements were performed as described previously [30].

### 4.7. Isothermal Titration Calorimetry (ITC) Analysis

ITC assays were performed on a Nano ITC Low Volume isothermal calorimeter (TA Instruments, New Castle, DE, USA) according to a previously described procedure [31]. XylR was dialyzed in the buffer (20 mM Tris-base, 100 mM NaCl, 5 mM MgCl_2_, and pH 7.5). All buffers were sonicated before use for degassing. XylR (35 μM) and the D-xylose or L-arabinose (300 μM) were added to the sample cell (190 μL) and the syringe (50 μL), respectively. There were 20 injections per experiment, and the stirring rate was 250 rpm. Additionally, the data were recorded automatically and subsequently analyzed by the NanoAnalyze Software (TA Instruments, New Castle, DE, USA). In control experiments, the D-xylose solution was titrated into the buffer in sample cells to obtain the heat of dilution. The value of the heat of dilution was then subtracted from the experimental curve in the final analysis. All the titration curves were fitted to the independent-site binding model.

### 4.8. Transcriptomic Analysis

*M. smegmatis* recombinant strains were cultured in 7H9 medium with shaking at 160 rpm at 37 °C until the mid-logarithmic phase. Then, the cells were harvested and washed with PBS. After that, transcriptomic analysis was performed, as described previously [32]. The red and blue spots represent the up-regulated and down-regulated genes, respectively, and the gray spots represent genes with insignificant change in expression.

### 4.9. GC/MS Assays for Detecting the Lipids of M. smegmatis

*M. smegmatis* recombinant strains were grown in 7H9 medium until mid-logarithmic phase. The cells were harvested and washed twice in PBS buffer. Extraction of lipids was performed according to the procedures previously [33]. GC/MS analyses were carried out according to the procedures previously with modifications [34]. The experiments were performed on a gas chromatograph Agilent 7890A coupled to Agilent 5975C mass spectrometer (Agilent Technologies, Santa Clara, CA, USA) using HP-5MS column (5% Phenyl Methyl Silox 30 m × 250 μm × 0.25 μm). The GC conditions were as follows: the injection volume was 1 μL and the flow rate of N_2_ as the carrier gas was 1.0 mL/min. For the temperature program, the initial temperature was 121 °C (hold 7 min), with a ramp to 170 °C at 4 °C/min (hold 3 min) and a ramp to 250 °C at 3 °C/min (hold 5 min), and the injector temperature was 280 °C.

### 4.10. Morphological Observation of Different Strains

*M. smegmatis* recombinant strains were grown in 7H9 medium until mid-logarithmic phase. After they were collected, the bacteria were washed with ddH_2_O to remove the remaining medium. The sample was suspended with appropriate amount of ddH_2_O, and the OD_600_ of the sample was kept at about 0.3. Then, 1 μL of the suspended substance was taken and coated on a clean slide. After being heated and fixed, the sample was stained with 1% crystal violet for 2 min. After that, the sample was cleaned and dried, and the colony morphology was observed under an optical microscope.

### 4.11. Observation of Bacterial Morphology through Scanning Electron Microscope

*M. smegmatis* recombinant strains with mid-logarithmic phase were collected and washed with ddH_2_O. The cells were fixed with 2.5% glutaraldehyde overnight and dehydrated with 30%, 60%, and 90% ethanol for 3–5 min. Then, the strains were collected and freeze-dried. Finally, the freeze-dried sample were glued and sprayed with gold for observation.

### 4.12. Observation and Quantification of Biofilm

*M. smegmatis* recombinant strains with mid-logarithmic phase were collected and resuspended in M63 medium. Then, the initial concentration was controlled to OD_600_ = 0.1. After 1.5 days, the biofilm was observed, the medium was drained, and the remaining biofilm was washed with ddH_2_O. After that, the biofilm was stained with 1% crystal violet and washed. Subsequently, ethanol/acetone mixture was added and kept at room temperature for 5 min. Finally, the absorbance was measured at 570 nm.

### 4.13. Morphological Observation and Determination of Mycobacterial Growth Curves

Strains were grown overnight in 7H9 medium at 37 °C with 160 rpm shaking. The cells were cultured until mid-logarithmic phase (OD_600_ ≈ 1.0) and 2 µL aliquots were spotted onto 7H10 medium containing 0.5% glycerol. The plates were incubated at 37 °C for 2–3 days. Colonies were photographed using a digital camera by macro shooting. To determine mycobacterial growth curves and the effects of antibiotics and D-xylose, each culture was diluted (4:100) in 100 mL of fresh 7H9 broth containing the indicated concentration of each antibiotic and D-xylose. The cultures were then allowed to grow further at 37 °C with shaking at 160 rpm. Aliquots were taken at the indicated times.

### 4.14. Intracellular Survival Assays

BMDMs were obtained by flushing the tibia and femurs of mice, as described previously [35]. The cells were cultured for five days at 37 °C [36]. The BMDMs cells were seeded in 24-well plates and cultured overnight. Then, the cells were infected with *M. bovis* BCG recombination strains at a multiplicity of infection of 10, as described previously [35]. At 4 hpi, the macrophages were washed thrice with PBS and were then added to the well-mixed medium. After incubation for 2–30 h, the cells were lysed using SDS, and diluted for plating on plates for counting cfu 15–21 days later. Penicillin/streptomycin was added to the medium, except during infections.

### 4.15. Mouse Infection

The female SPF C57BL/6 mice weighed 16–18 g and they were 6–8 weeks old during the experimental period. The mice were housed in a specific pathogen-free facility that used standard humane animal husbandry protocols, which were approved by the Animal Experimental Ethics Committee of Guangxi University on 8 March 2021 (Gxu-2021-073). *M. bovis* BCG recombination strains were cultured until mid-logarithmic phase (OD_600_ ≈ 1.0) and washed three times with PBS containing 0.05% Tween 80. The 36 mice were randomly divided into six groups (*n* = 6), and were intratracheally infected with 1 × 10^6^ cfu of BCG/pMV261 and BCG/pMV261-*xylR* strains separately. The results were analyzed as described previously [37].

## 5. Conclusions

In conclusion, we found that D-xylose could act as a signaling molecule to block the broad negative regulation of XylR on lipid metabolism. In addition, D-xylose affected multiple physiological characteristics in mycobacteria, including bacterial size, colony phenotype, biofilm formation, cell aggregation, and antibiotic resistance. Finally, we found that XylR inhibited the survival of *M. bovis* BCG in the host. Our findings provide new insights into the correlation between lipid metabolism regulation mechanisms and bacterial physiological phenotypes.

## Data Availability

The transcriptomic data of *M. smegmatis* were deposited to the NCBI Sequence Read Archive (SRA) database (SRA accession number: PRJNA923393).

## Supplementary Materials

The following supporting information can be downloaded at: https://www.mdpi.com/article/10.3390/ijms24087086/s1.
