# Supplementary material for: D-Xylose Blocks the Broad Negative Regulation of XylR on Lipid Metabolism and Affects Multiple Physiological Characteristics in Mycobacteria
Source: Int J Mol Sci. 2023 Apr 11;24(8):7086. doi: 10.3390/ijms24087086 (PMC10138657; doi:10.3390/ijms24087086)
Supplement: Supplementary file 1 [file ijms-24-07086-s001.zip › Supplementary Data.pdf]

D-Xylose Blocks the Broad Negative Regulation of XylR on Lipid Metabolism and Affects Multiple Physiological Characteristics in Mycobacteria

Kun Wang †, Xujie Cui †, Xiaocui Ling, Jiarui Chen, Jiachen Zheng, Yuling Xiang and Weihui Li *

State Key Laboratory for Conservation and Utilization of Subtropical Agro-Bioresources, College of Life Science and Technology, Guangxi University, Nanning 530004, China; 2008401012@st.gxu.edu.cn (K.W.); 2208391004@st.gxu.edu.cn (X.C.); lingxiaocui2017@163.com (X.L.); chenjiarui@genomics.cn (J.C.); zjc13737366820@163.com (J.Z.); xyl9724@163.com (Y.X.)

* Correspondence: lwhlbx@163.com; Tel.: +86-771-285-2965

† These authors contributed equally to this work.

A list of the supplemental materials:

Figure S1. Bacterial one-hybrid assay for XylR and *xylR*p interaction.

Figure S2. EMSA assays for XylR binding to DNA substrates of mutant IR sequence.

Figure S3. The assays of β-galactosidase activity.

Figure S4. ITC assays for interaction between XylR and L-Ara.

Figure S5. The assays for the growths of wildtype and recombinant *M. smegmatis* strains under the induction of D-xylose.

Table S1. Transcriptomic assays for the differential gene expression of the *xylR*-overexpressing compared to the Msm/pMV261 strain (provided as an Excel table, separately)

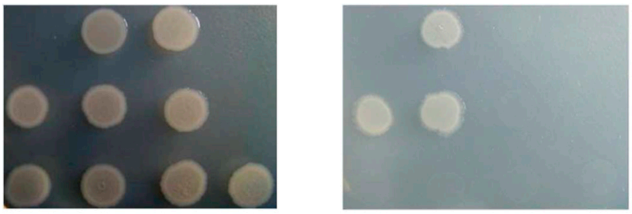

-3AT -Str^{r} +3AT +Str^{r}

	CK^{+}	CK^{-}	
pTRG-*xylR*/ pBX-*Ms6021*p	pTRG-*xylR*/ pBX-*xylR*p	pTRG-*xylR*/ pBX-*Ms6020*p	
pTRG/ pBX-*Ms6021*p	pTRG/ pBX-*xylR*p	pTRG/ pBX-*Ms6020*p	pTRG-*xylR*/ pBXcmT

Figure S1. Bacterial one-hybrid assay for XylR and *xylR*p interaction. A pair of pBXcmT/pTRG plasmids was co-transformed into the reporter strain and its growth was monitored together with that of the self-activation controls on selective medium. Co-transformants containing the pBX-*Rv2031*/pTRG-*Rv3133c* plasmids served as positive controls (CK^{+}) and co-transformants containing the empty vectors pBX and pTRG served as negative controls (CK^{-}).

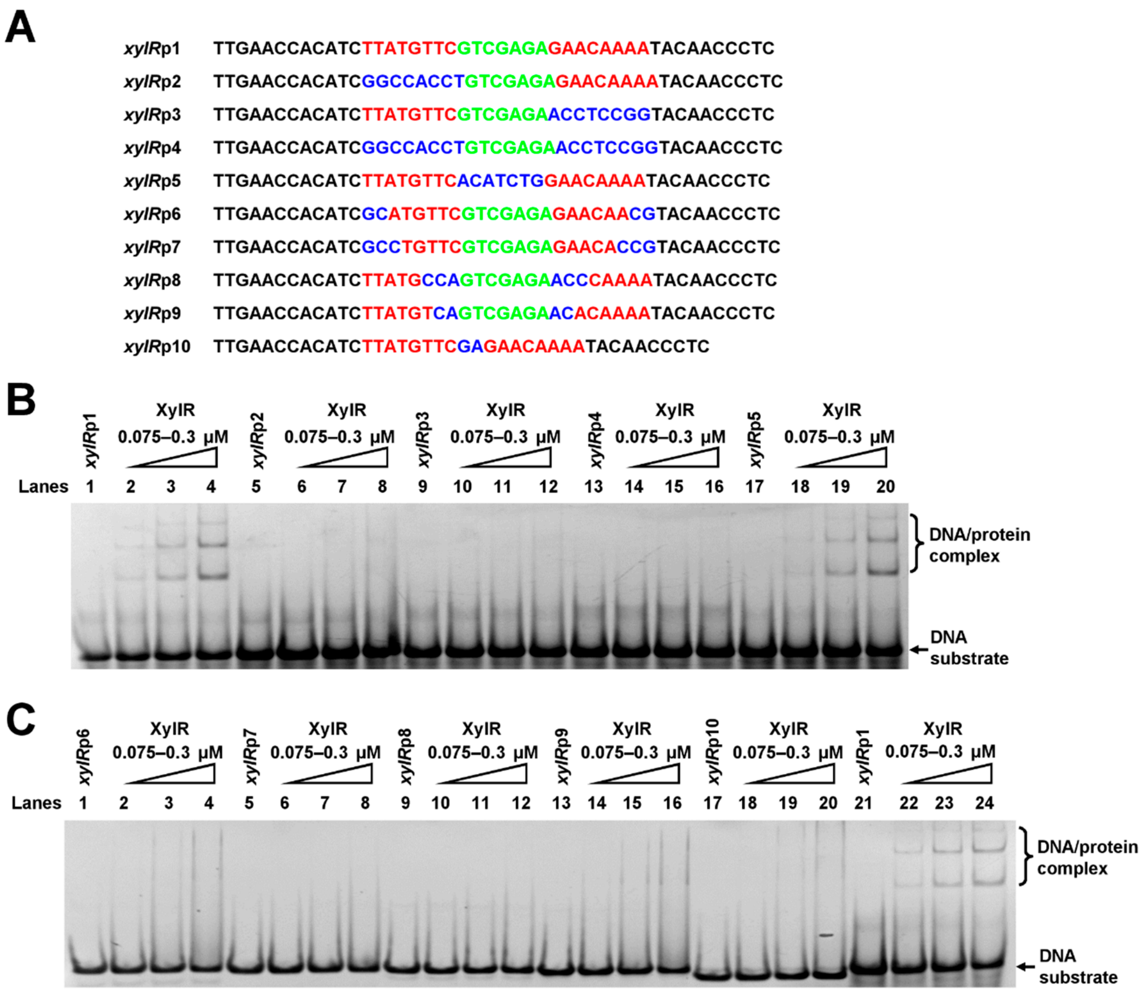

Figure S2. EMSA assays for XylR binding to DNA substrates of mutant IR sequence. **(A)** The wild-type IR sequence (*xylR*p1) and mutated sequences (*xylR*p2-p10) were listed. The DNA-binding activity of XylR on wild-type IR sequence (lanes 1-4) **(B)** and mutated sequences (lanes 5-20) **(B)**, (lanes 1-20) **(C)** were shown in figures. The DNA substrates were co-incubated with 0.075-0.3 μM of XylR protein.

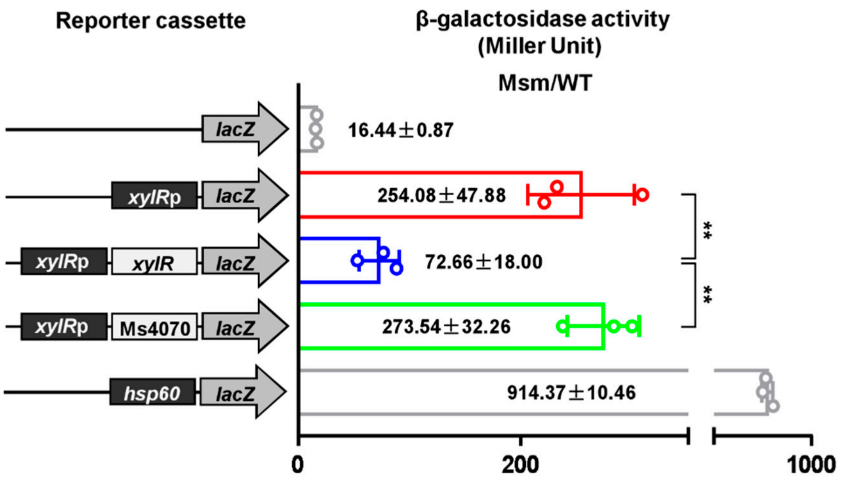

Figure S3. The assays of β-galactosidase activity. The effect of XylR on the gene expression was assayed by constructing a series of *lacZ* alone or promoter-*lacZ* and promoter-*xylR*-*lacZ* co-expression plasmids. These plasmids were transformed into Msm/WT strains. The activity of β-galactosidase was further examined and presented as Miller units (right panel). Left column: schematic representation of each clone used to generate recombinant strains. Null promoter-*lacZ*, *xylR*p-*Ms4070*-*lacZ* and *hsp60*-*lacZ* were used as controls. Right column: β-galactosidase activity was expressed as Miller units. The values presented were the averages of three independent experiments. For statistical analysis, two-way analysis of variance with Bonferroni multiple comparison tests were performed using a *P*-value of < 0.01. The *P*-values of the results (<0.01) are indicated by two asterisk (**) on top of the column in the figure.

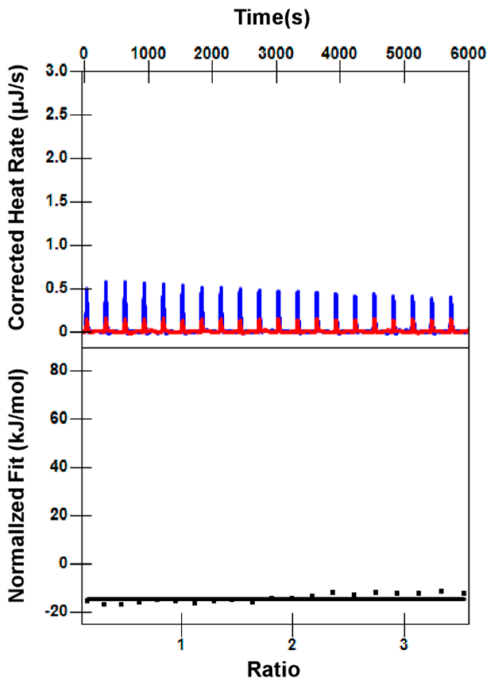

Figure S4. ITC assays for interaction between XylR and L-Ara. Original titration data and integrated heat measurements are shown in the upper and lower plots, respectively.

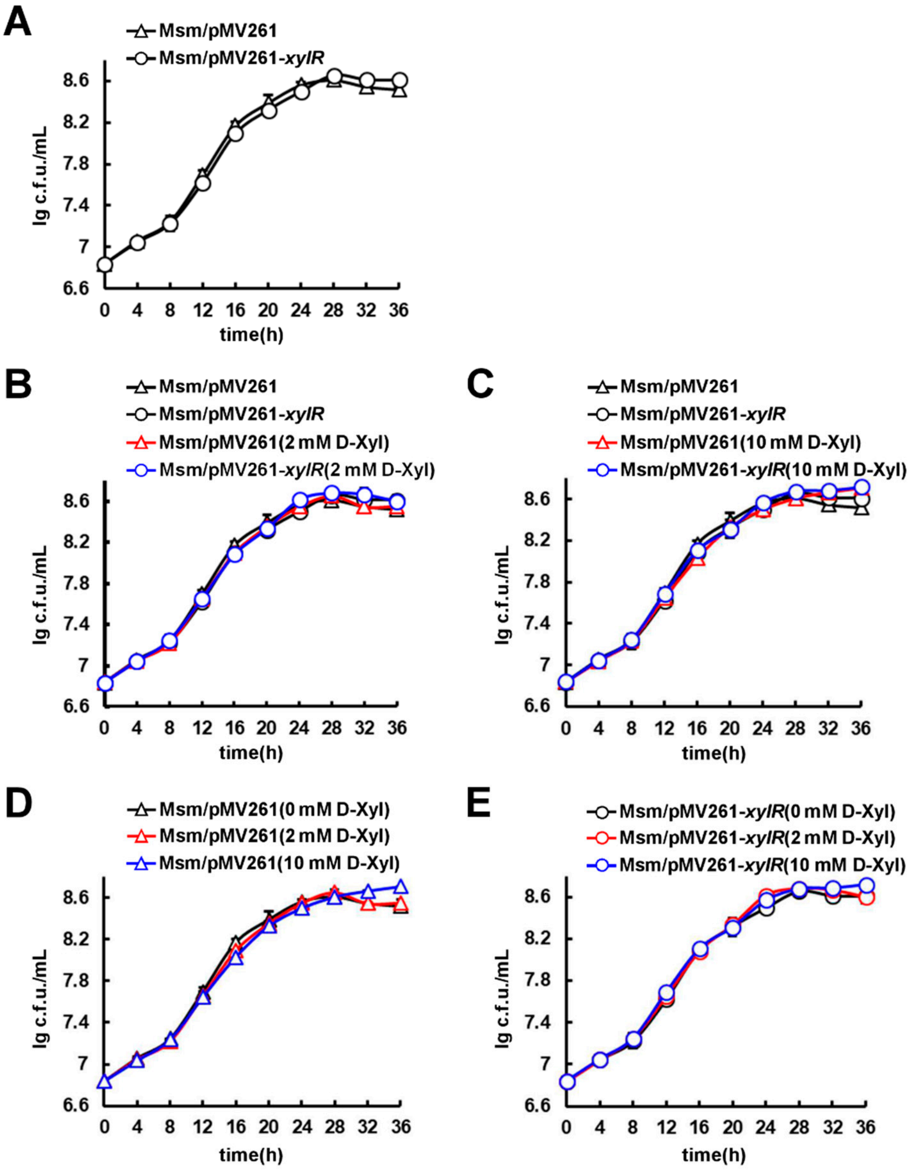

Figure S5. The assays for the growths of wildtype and recombinant *M. smegmatis* strains under the induction of D-xylose. Growth curves of the recombinant *M. smegmatis* strains were determined as described in 'Materials and Methods' section. The *xylR*-overexpression and wild-type strains **(A)** were grown in 7H9 medium without antibiotic stress and no obvious growth difference was observed. When 2 or 10 mM D-xylose was added into the medium as a stress inducer, no growth difference between the wild type *M. smegmatis* and *xylR*-overexpression strains **(B-E)**.
